# Supplementary material for: The interactome of intact mitochondria by cross-linking mass spectrometry provides evidence for coexisting respiratory supercomplexes
Source: Mol Cell Proteomics. 2017 Dec 8;17(2):216–32. doi: 10.1074/mcp.RA117.000470 (PMC5795388; doi:10.1074/mcp.RA117.000470)
Supplement: Supplemental Data [file supp_RA117.000470_133922_0_supp_23978_fzffwf.zip › spectra_annotation/mito_spectra_annotation/367-1-6-1-5-1.pdf]

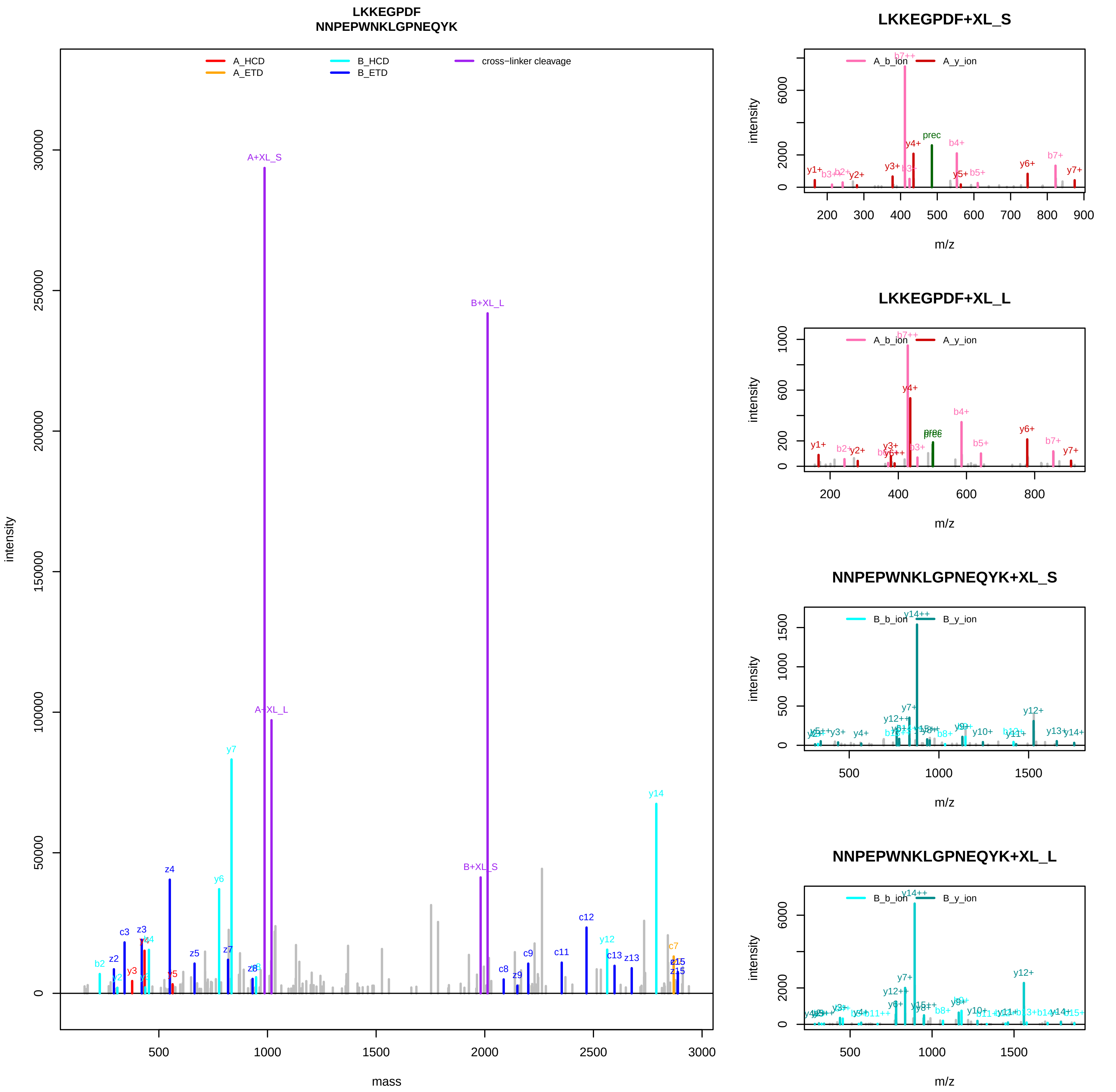

LKKEGPDF
NNPEPWNKLGPNEQYK
A_HCD
A_ETD
B_HCD
B_ETD
cross−linker cleavage
A+XL_S
B+XL_L
A+XL_L
B+XL_S
y7
y6
y14
y12
z4
c3
z3
z2
b2
y3
y5
z5
z7
z8
c7
z15
c8
z9
c9
c11
c12
c13
z13
intensity
mass
0
50000
100000
150000
200000
250000
300000
500
1000
1500
2000
2500
3000
LKKEGPDF+XL_S
A_b_ion
A_y_ion
b7++
prec
y4+
b4+
y3+
y6+
b7+
y7+
y1+
y2+
y5+
b5+
intensity
m/z
LKKEGPDF+XL_L
NNPEPWNKLGPNEQYK+XL_S
B_b_ion
B_y_ion
y14++
y7+
y12++
y12+
y10+
y13+
y14+
y3+
y4+
b8+
NNPEPWNKLGPNEQYK+XL_L
y15++
y9+
y11+
